# Using Coexpression Protein Interaction Network Analysis to Identify Mechanisms of Danshensu Affecting Patients with Coronary Heart Disease

**DOI:** 10.3390/ijms18061298

**Published:** 2017-06-19

**Authors:** Mengqi Huo, Zhixin Wang, Dongxue Wu, Yanling Zhang, Yanjiang Qiao

**Affiliations:** Key Laboratory of Traditional Chinese Medicine Information Engineer of State Administration of Traditional Chinese Medicine; School of Chinese Material Medica, Beijing University of Chinese Medicine, Beijing 100102, China; 20150931715@bucm.edu.cn (M.H.); wangzx@bucm.edu.cn (Z.W.); 18810820682@163.com (D.W.)

**Keywords:** Danshensu (DSS), coronary heart disease (CHD), targets, coexpression protein interaction network (CePIN)

## Abstract

*Salvia miltiorrhiza*, known as Danshen, has attracted worldwide interest for its substantial effects on coronary heart disease (CHD). Danshensu (DSS) is one of the main active ingredients of Danshen on CHD. Although it has been proven to have a good clinical effect on CHD, the action mechanisms remain elusive. In the current study, a coexpression network-based approach was used to illustrate the beneficial properties of DSS in the context of CHD. By integrating the gene expression profile data and protein-protein interactions (PPIs) data, two coexpression protein interaction networks (CePIN) in a CHD state (CHD CePIN) and a non-CHD state (non-CHD CePIN) were generated. Then, shared nodes and unique nodes in CHD CePIN were attained by conducting a comparison between CHD CePIN and non-CHD CePIN. By calculating the topological parameters of each shared node and unique node in the networks, and comparing the differentially expressed genes, target proteins involved in disease regulation were attained. Then, Gene Ontology (GO) enrichment was utilized to identify biological processes associated to target proteins. Consequently, it turned out that the treatment of CHD with DSS may be partly attributed to the regulation of immunization and blood circulation. Also, it indicated that sodium/hydrogen exchanger 3 (SLC9A3), Prostaglandin G/H synthase 2 (PTGS2), Oxidized low-density lipoprotein receptor 1 (OLR1), and fibrinogen gamma chain (FGG) may be potential therapeutic targets for CHD. In summary, this study provided a novel coexpression protein interaction network approach to provide an explanation of the mechanisms of DSS on CHD and identify key proteins which maybe the potential therapeutic targets for CHD.

## 1. Introduction

Coronary heart disease (CHD) is a leading cause of morbidity and mortality globally. It is a common disease associated with vascular stenosis or obstruction caused by coronary artery vascular lesions [1]. *Salvia miltiorrhiza*, an established traditional Chinese medicine (TCM) with 1000 years of clinical application, can promote blood circulation and eliminate blood stasis [2,3]. It has also been extensively used in the treatment of CHD. Danshensu (DSS) is the main effective monomer component isolated from root of *Salvia miltiorrhizae* [4]. Its pharmacological effect include the following: protect the myocardium [5]; inhibit platelet aggregation [4]; anti-inflammatory [6]; anti-atherosclerosis [7], and; anti-thrombosis [8], among others. These research findings explain the action mechanisms of DSS to some extent, but further study is still needed. In the current study, the mechanisms of DSS were illuminated by a coexpression protein interaction network (CePIN), which would probably be beneficial for the development of targets for CHD therapeutic intervention.

**Figure 1:**
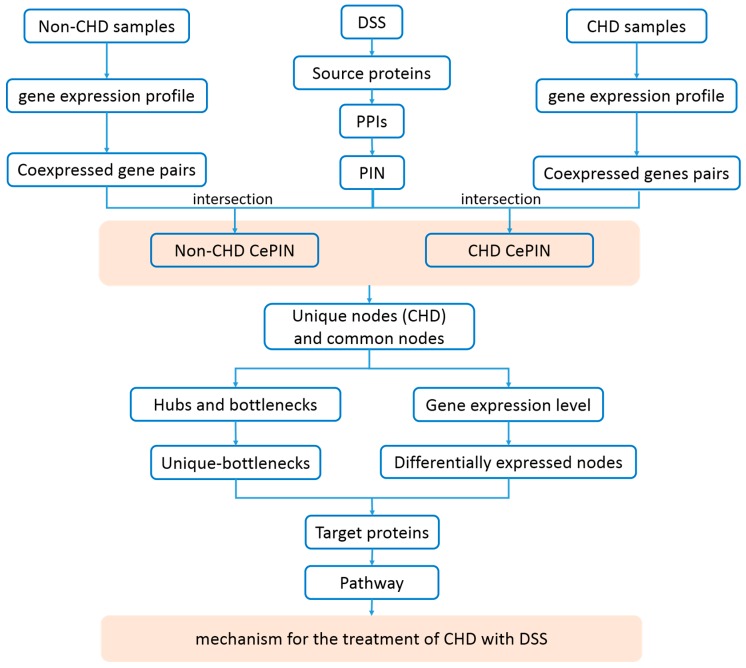
The whole framework of this study is based on a coexpression protein interaction network (CePIN) for the identification of mechanisms for the treatment of coronary heart disease (CHD) with Danshensu (DSS).

High throughput screening, computational prediction, text mining, etc., typically generate a large number of protein-protein interactions (PPIs) data [9]. These data, accumulated under different states and times, contain noisy data and false positives, which cannot be used directly to build a network [10]. Coexpression analysis is a method of analyzing the correlation between genes based on one state, which removes noisy data, and also finds the core nodes of network by calculating the network topology to analyze the interactions between nodes [11]. In addition, differentially expressed genes are likely to ultimately control the morphological and physiological processes of the organism [12]. Therefore, the analysis of differentially expressed genes is beneficial to identify the core genes that regulate the process of cell life. Although the expression level of the gene hardly represents the concentration of proteins, a significant correlation between them has been proved [13], and the genetic data can indirectly reflect the relationship between proteins. In this case, it is necessary to integrate gene expression data, so as to leave the really significant proteins, which is equivalent to constructing CePIN in different states [14,15]. A comparison of network topology parameters and differentially expressed genes in different states is undertaken to elucidate the mechanisms of treatment of CHD by DSS. In the current study, by integrating gene expression profile data and PPI of DSS, the coexpression protein interaction networks (CePIN) in a CHD state (CHD CePIN) and non-CHD state (non-CHD CePIN) were constructed, respectively. Based on the calculation of the topological parameters and the analysis of differentially expressed genes in the two states, target proteins can be obtained. Gene Ontology (GO) enrichment analysis was used to determine the metabolic pathway involved in these targets. The results can provide an efficient way to elucidate the mechanisms of DSS as anti-CHD agents and determine the potential targets based on an analysis of CePIN. The experimental flow chart is shown in Figure 1.

## 2. Results

### 2.1. Source of Protein Information Related to Danshensu (DSS)

Eight proteins were obtained from pharmacophore-based virtual screening, and one protein was extracted from STITCH database (version 4.0, Available online: http://stitch.embl.de/). Proteins information of DSS was shown in Table 1.

### 2.2. Construction of Coexpression Protein Interaction Networks (CePIN)

The protein-protein interactions (PPIs) information of proteins was obtained from the String database (version 9.1, Available online: http://string-db.org/) with the confidence score > 0.7, and then was imported in Cytoscape; the Union calculation was carried out, followed by the removal of duplicated edges of PPIs using the Advanced Network Merge. The protein interaction network (PIN) of DSS was shown in Figure S1.

In the microarray data, the coexpression is reflected by strong correlations between expression levels, while not all changes in coexpression are manifested by up- or down-regulation of individual genes [11,16,17,18]. In order to guarantee the completeness of the data, all genes were used to calculate the correlation. Details of the microarray data were shown in Tables S1 and S2.

**Figure 2:**
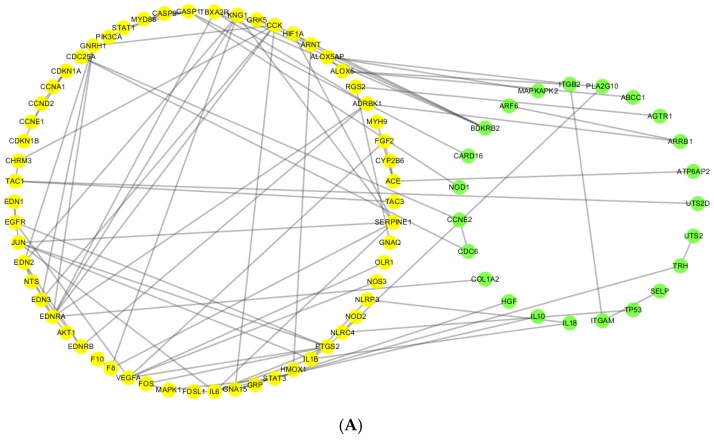

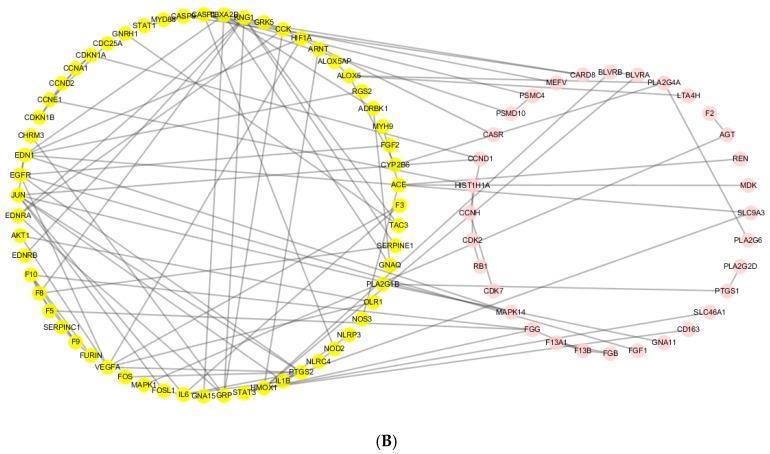
(**A**) Coexpression protein interaction networks (CePIN) in a non-CHD state (non-CHD CePIN) and (**B**) coexpression protein interaction networks (CePIN) in a CHD state (CHD CePIN). Nodes in green color are unique nodes in non-CHD CePIN; those in yellow color are shared nodes both in non-CHD CePIN and CHD CePIN; those in pink color are unique nodes in CHD CePIN; and the blacklines are interactions between nodes.

All coexpression protein-protein interactions (CePPIs) were imported into Cytoscape software to create networks. As shown in Figure 2, non-CHD CePIN contained 91 nodes and 98 edges, and CHD CePIN contained 99 nodes and 110 edges. Details of protein information of CHD CePIN and non-CHD CePIN shown in Table S3.

### 2.3. Comparative Analysis of CePIN 

The shared nodes and the unique nodes in CHD CePIN were identified by comparing CHD CePIN and non-CHD CePIN; the results were shown in the Table S1 and Figure 2.

A structural analysis of CePIN was shown in Table 2. The overlap rate of proteins in two CePIN was 69%, but the overlap rate of CePPIs was only 32%. It suggested that most of protein interactions between the two states were different. 

#### 2.3.1. Comparative Analysis of Topological Parameters of Proteins in CePIN

**Figure 3:**
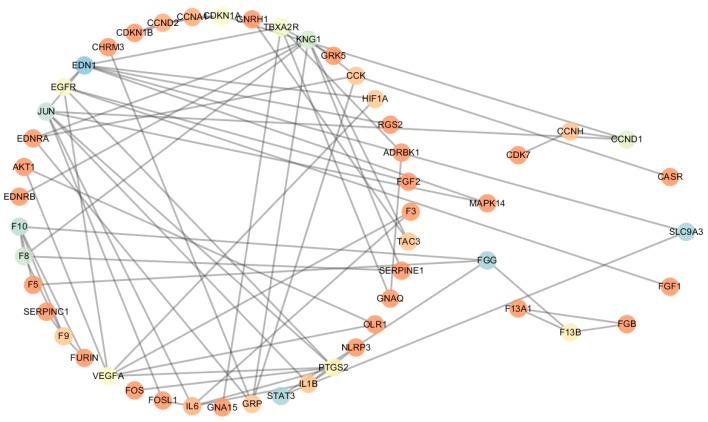
CHD CePIN colored based on the betweenness of each node. Red (least) → light red → yellow → light blue → blue (greatest). Shared nodes are on the left, and unique nodes are on the right.

In order to describe the global characteristics of CHD CePIN, the topological parameters were firstly elucidated in Table 3, Table S4 and Figure 3 (The topological parameters of non-CHD CePIN were elucidated in Figure S2). Endothelin-1 (EDN1) was a hub-bottleneck in CHD CePIN, which owned the highest values of degree and betweenness. Fibrinogen gamma chain (FGG), Sodium/hydrogen exchanger 3 (SLC9A3) and signal transducer and activator of transcription 3 (STAT3) were all identified as bottlenecks with the values of betweenness higher over threshold (+2 SD). Moreover, both transcription factor AP-1(JUN) and Kininogen-1(KNG1) were regarded as hubs with their degree over corresponding thresholds.

According to the results of aforementioned topological analysis, these six nodes were initially used as the candidate targets. After the removal of candidate targets, the topological parameters of CHD CePIN were shown in Table 4.

As shown in Table 4, after deleting the candidate targets, the network diameter, the shortest path, and characteristic path length have significantly greater changes. The network diameter is the largest distance between two nodes. If a network is disconnected, its diameter is the maximum of all diameters of its connected components [19]. The shortest path, also called distance, refers to the distance between two nodes in the network [20]. The characteristic path length, known as the average shortest path length, is the average of the shortest path lengths between all pairs of nodes in the network [21]. They all reflect the connectivity of biological networks [19,22,23]. After deleting the six candidate targets, the above parameters were significantly decreased, and the entire network would be decomposed into small components. It is worth mentioning that a greater change has taken place in the network after deleting the bottlenecks. It suggested that the bottlenecks in CHD CePIN may play a more prominent role.

In a previous study [24], we always paid more attention to hubs which could play key roles in the network. However, when we calculated topological parameters of nodes in CHD CePIN for identifying potential drug targets, we inevitably found many problems. There were some hubs with high values of degree in CHD CePIN, but this did not mean that they were the best targets for CHD treatment. These proteins may also exist and play important roles in non-CHD CePIN networks. Based on our current results, we found that hubs often existed in shared nodes. That is to say, these hubs may be involved in more physiological activities and play prominent roles both in CHD and non-CHD state. Moreover, there were some bottlenecks in CHD CePIN, which were always ignored due to low values of degree. In fact, the regulation of bottlenecks would seriously impact the entire network more than hubs. Were the bottlenecks removed, the entire network would be decomposed into small components. It was worth mentioning that some bottlenecks (FGG, SLC9A3) may have better efficacy for CHD, because they were unique nodes in CHD CePIN. When these proteins were disturbed, CHD CePIN would be significantly affected, but non-CHD CePIN would not be affected. Thereby, FGG and SLC9A3 may be key proteins for DSS to play a potential role as a therapeutic strategy. Beyond this, the design of drugs for these two targets may reduce the side effects. The results identified the unique-bottlenecks (unique-bottleneck refers to a node that is both a unique node and a bottleneck) FGG and SLC9A3 as the targets of DSS in the treatment of CHD, which were also potential targets for new drug design.

#### 2.3.2. Comparative Analysis of the Expression Level of the Gene Corresponding to the Proteins in CePIN

Based on the LIMMA package (a software package for the analysis of gene expression studies), prostaglandin G/H synthase 2 (PTGS2) and oxidized low-density lipoprotein receptor 1 (OLR1) were identified with logFC −1.61 and −1.55, respectively (see Table S5). The results showed that the gene expression levels of these nodes were significantly different. Therefore, PTGS2 and OLR1 may be targets of DSS in the treatment of CHD and played different roles in the two states.

### 2.4. Gene Ontology (GO) Enrichment Analysis of CePIN

**Figure 4:**
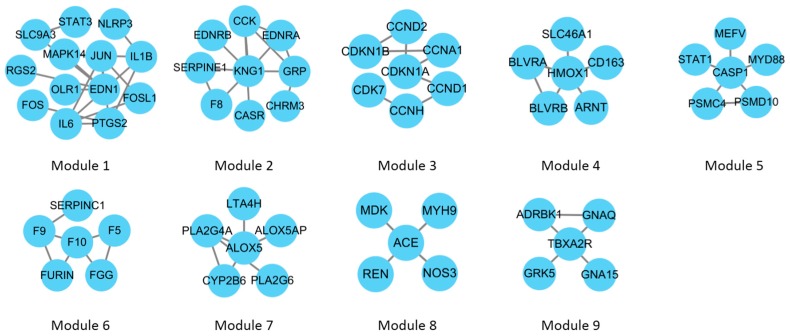
Modules in CHD CePIN. With the fast agglomerate algorithm based on the edge clustering coefficients (FAG-EC) algorithm, nine modules were extracted from the network.

Function modules of CHD CePIN were clustered by using a fast agglomerate algorithm based on the edge clustering coefficients (FAG-EC), and nine modules were identified (see Figure 4). The main biological processes involved in each module were shown in Table 5. SLC9A3, PTGS2 and OLR1 were involved in inflammatory response, and FGG were involved in blood coagulation.

## 3. Discussion

In summary, this work proposed a novel method of elucidating the action mechanisms of DSS on CHD by integrating the gene expression profile data and PPIs. A comparison of CePIN in different states was used to identify potential therapeutic targets (FGG, SLC9A3, PTGS2 and OLR1). In addition, inflammatory response and blood coagulation were also indicated as the main mechanism in CHD treatment by DDS. However, further studies are required to determine the clinical utility of these observations in the therapeutic management of CHD. 

According to our results, FGG and SLC9A3 were unique-bottlenecks in CHD CePIN, which represented two optimal therapeutic targets to be tested in vitro and/or in vivo. SLC9A3 was related to the inflammatory reaction mediated by T cells, its expression and activity were significantly inhibited in vitro by interferon gamma [25]. Meanwhile, inflammation was an independent risk factor, which can accelerate the atherosclerotic process to aggravate CHD [26]. Therefore, SLC9A3 may regulate CHD by participating in the inflammatory response. In addition, some research findings suggested that the fibrinogen level and genetic variation in the *FGG* gene may influence arterial stiffness, which can be used to predict cardiovascular disease [27]. Furthermore, PTGS2 and OLR1, two shared nodes, had changed significantly and played different roles in two states, and both of them were potential therapeutic targets. There were some pharmacological experiments that proved that PTGS2 and OLR1 can increase the risk of CHD [28,29]. All targets above were directly or indirectly related to CHD. However, there were still few reports about the treatment of CHD with these targets by DSS, and the study on treating CHD with DSS was still at an exploratory stage. These four targets are likely to be potential therapeutic targets of treatment of CHD with DSS. 

It was possible that some other unique nodes in CHD CePINs may also play a vital role in the regulation of CHD, despite their topological parameters that did not reach the thresholds. Typically, CCND1, F13B, CCNH, MAPK14, FGF1, FGB, F13A1, CDK7, and CASR were directly or indirectly linked with therapeutic targets, and they may be involved in the regulation of CHD in other ways. Currently, there have been continuous attempts in the regulation of these proteins on CHD. As reported in the literature, MAPK14 was related to myeloperoxidase, which was considered as a prognostic cardiovascular risk marker [30]. FGF1 can promote cardiac regeneration in a myocardial infarction rat model [31]. FGB polymorphism contributed to the development of CHD [32]. The Xueshuan Xinmaining Tablet ameliorated blood stasis by regulating the expressions of F13A1 [33]. Serine at position 986 of CASR may be an independent genetic predictor of angiographic coronary artery disease [34]. These proteins also can be utilized as potential drug targets for CHD treatment in the future, but still require further research. 

In the current study, the mechanisms of DSS treating CHD were elucidated to some extent, but the results need to be analyzed and validated by later cell or animal experiments. For the treatment of CHD, there is still a demand for continued researches and development of new drugs. With the aid of CePIN, it is possible to analyze multiple candidate targets and biological processes within a network model simultaneously. However, there are some shortcomings in this study. The data set used in this research is very small and further experiments are needed to confirm the conclusions. Omics data found in publicly available databases (e.g., ChEMBL, Reactome, or KEGG) can be used to validate our results or further expand the protein interactions data. Finally, this method is not limited to the study of DSS. Additionally, it can be used as an objective tool to identify, with measurable parameters, candidate targets to treat other diseases and discover potential targets for other drugs. 

## 4. Materials and Methods

### 4.1. Construction of Protein Interaction Networks (PIN)

The information of source proteins of DSS was obtained from two sources: one was the STITCH database (version 4.0, Available online: http://stitch.embl.de/) [35], and another was a pharmacophore-based virtual screening. The STITCH database is an open source database of protein-chemical interactions that integrates lots of information of experimental and manually curated evidence with text-mining and interaction predictions, and has a score for each pair of protein-chemical interactions relationship. The proteins with high confidence (confidence score > 0.7) were chose to ensure data reliability [36].

Based on more than 100 pharmacophore models constructed previously by our laboratory [37,38,39,40,41], the ones matched with DSS were searched and their Fit values compared to the chemical structure of DSS were calculated. The proteins corresponding to pharmacophore models with Fit value >0.7 were selected as source proteins used in the following study [42]. 

The PPIs of source proteins were obtained from String database (version 9.1, Available online: http://string-db.org/) [43] which provided score for evaluating the relevance between any two inter-actors. Those PPIs with a confidence score >0.7 were applied to construct PIN of DSS by Cytoscape software [44].

### 4.2 Construction of CePIN

The CHD gene expression profile GSE42148 (Gene Expression Omnibus (GEO) accession number) was downloaded from GEO database [45] (Available online: http://www.ncbi.nlm.nih.gov/geo/), which was based on the platform of GPL13607 Agilent-028004 SurePrint G3 Human GE 8x60K Microarray (David Packard and William Redington Hewlett, California, USA).

The microarray data came from 13 patients with angiographically confirmed CHD between ages 40–55 years and 11 population-based asymptomatic controls with normal ECG and matched for age, gender and common risk factors such as diabetes and hypertension to that of the cases. Global gene expression profiling was performed on the Agilent microarray platform. The original microarray data often needs to be balanced and modified before the subsequent analysis. The scanned images were analyzed with Feature Extraction Software 10.7.3.1 (Agilent, David Packard and William Redington Hewlett, California, USA) using default parameters (protocol GE1_107_Sep09 and Grid: 028004_D_F_20101102) to obtain background subtracted and spatially detrended Processed Signal intensities. Features flagged in Feature Extraction as Feature Non-uniform outliers were excluded. 

The Pearson correlation coefficient was adopted to determine whether some genes were coexpressed in one state. The Pearson correlation coefficient (*r*) of genes was represented as follows:

(1) $$r=\frac{1}{n-1}\sum_{i=1}^{n}\left(\frac{X_{i}-\bar{X}}{S_{X}})(\frac{Y_{i}-\bar{Y}}{S_{Y}}\right)$$

where *n*, $X_{i}$($Y_{i}$), $\bar{X}$($\bar{Y}$) and $S_{X}$($S_{Y}$) are sample number, expression level of gene X(Y) of sample i, average expression level of gene X(Y), and standard deviation of expression level of gene X(Y) in a given state. As the absolute value of *r* gets larger, the relationship between two related genes gets closer.

Coexpressed genes pairs in one state can be obtained from the above formula. When the expression level of corresponding coding genes was related, the interacting proteins were significantly related. With the correlation coefficient |*r*| greater than 0.7, the coexpressed genes were selected (*p* < 0.05) [46]. CePPIs of each state could be obtained by the intersection of coexpressed genes pairs and PIN. CePPIs were imported into Cytoscape software to create visualized networks. All CePPIs in a disease state were used to constitute CHD CePIN, while all CePPIs in a normal state were used for non-CHD CePIN.

### 4.3. Comparative Analysis of CePIN

In order to reveal the changes of proteins of CePIN, CHD CePIN and non-CHD CePIN were compared to identify shared nodes and the unique nodes in CHD CePIN.

The structural changes of CePIN were revealed by comparing CePPIs in different states. Overlap rates of proteins and CePPIs in two states were calculated by Cytoscape.

#### 4.3.1. Comparative Analysis of Topological Parameters of Proteins in CePIN

The following measures were taken to calculate the topology parameters of the nodes in the network: (1) Disconnected nodes and edges were deleted to acquire the maximal connected subset of CHD CePIN. (2) The Network Analyzer plugin for Cytoscape software was employed to compute a set of comprehensive topological parameters of all nodes in networks, including degree and betweenness, etc. Degree means the number of edges linked to a given node (in undirected networks). Nodes with high values of degree over the thresholds values are named as “hubs”. In scale-free CePINs, most proteins interact with few partners, while a small but significant proportion of proteins, which are also called “hubs”, interact with a large number of partners [47,48]. CePINs are particularly resistant to the removal of non-hubs, but extremely sensitive to the targeted removal of hubs. Therefore, the removal of hubs leads to paralysis and breakdown of network, which affected the connectivity of the whole network [49]. For instance, the knockout of genes encoding hubs brings about approximately threefold lethality than the knockout of non-hubs [47]. Generally speaking, hubs are more likely to be key nodes and play a leading role in the regulation of networks [50]. In addition, betweenness represents the centrality of a node in a network, and is normally regarded as the fraction of shortest paths between node pairs that pass through a given node [51]. In a biological network, a node with higher betweenness will have greater influence on the network, because more interactions will pass through this node [52]. It indicated that those nodes are key points which control the communication with other nodes in CePIN. Were they removed, the network would be divided into fragments [53]. All nodes with high values of betweenness over threshold are named as “bottlenecks” [54]. Therefore, the above two parameters reflect the node’s influence on the network. Values of betweenness or degree above two standard deviation (+2 SD) compared with the mean were selected to identify candidate targets [55]. This research calculated the network connectivity to determine the targets in CHD CePIN after removing the candidate nodes [23].

#### 4.3.2. Comparative analysis of the expression level of the gene corresponding to the proteins in CePIN

Shared nodes played roles in the two states, which was difficult to assess just by topology. Therefore, analysis of differentially expressed genes was applied to show their difference between the two states. The LIMMA package (Available online: http://www.bioconductor.org/packages/release/bioc/html/limma) was utilized to identify differentially expressed genes, with *p* < 0.05 and |logFC| > 1 selected as the thresholds [56].

### 4.4. GO Enrichment Analysis of CePIN

The functional modules were enriched and analyzed to understand the biological processes involved in key nodes [57]. Functional modules of CePIN were first explored by the FAG-EC algorithm, which could be used for large-scale PIN and showed hierarchical structure of functional modules by modifying the parameters [58]. Then the identified modules were used for functional enrichment analysis by the BinGO plugin for Cytoscape. GO enrichment analysis was utilized to predict possible biological roles of the modules [59]. Gene annotation information of proteins was all from Gene ontology [60] (Available online: http://www.geneontology.org/).

## Figures and Tables

**Table 1:** Proteins information of Danshensu (DSS).

Uniprot ID	Proteins	Source	Uniprot ID	Proteins	Source
P09601	HMOX1	STITCH	P12821	ACE	Pharmacophore
P30556	AGTR1	Pharmacophore	P09917	ALOX5	Pharmacophore
P25101	EDNRA	Pharmacophore	P29466	CASP1	Pharmacophore
P24530	EDNRB	Pharmacophore	P00742	F10	Pharmacophore
P24941	CDK2	Pharmacophore	-	-	-

Heme oxygenase 1 (HMOX1); type-1 angiotensin II receptor (AGTR1); endothelin-1 receptor (EDNRA); endothelin B receptor (EDNRB); cyclin-dependent kinase 2 (CDK2); angiotensin-converting enzyme (ACE); arachidonate 5-lipoxygenase (ALOX5); caspase-1 (CASP1); coagulation factor X (F10).

**Table 2:** Structural analysis of coexpression protein interaction networks (CePIN).

Items	Proteins	CePPIs
CHD CePIN	91	98
Non-CHD CePIN	99	110
Overlap amount	66	33
Overlap ratio	69%	32%

**Table 3:** Nodes with the highest degree and betweenness values in CHD CePIN.

Name	Category	Hub/Bottleneck	Betweenness	Degree
EDN1	shared	hub-bottleneck	0.63673203 ^a^	7 ^b^
FGG	unique	bottleneck	0.51450980 ^a^	4
SLC9A3	unique	bottleneck	0.49411765 ^a^	2
STAT3	shared	bottleneck	0.48627451 ^a^	2
F10	shared	-	0.41058824	5
JUN	shared	hub	0.37490196	7 ^b^
F8	shared	-	0.32156863	4
KNG1	shared	hub	0.28313725	7 ^b^
CCND1	unique	-	0.21803922	3
TBXA2R	shared	-	0.15137255	5
Average	-	-	0.091432882	2.7307692
+1 SD	-	-	0.254025973	4.4810116
+2 SD	-	-	0.416619064	6.2312540

^a,b^ Refers to nodes with values of betweenness and degree above two standard deviations (+2 SD) compared with thresholds, resp. hub-bottleneck refers to a node both hub and bottleneck. Endothelin-1 (EDN1); fibrinogen gamma chain (FGG); sodium/hydrogen exchanger 3 (SLC9A3); signal transducer and activator of transcription 3 (STAT3); coagulation factor X (F10); transcription factor AP-1 (JUN); coagulation factor VIII (F8); kininogen-1 (KNG1); G1/S-specific cyclin-D1 (CCND1); thromboxane A2 receptor (TBXA2R); Average refers to average of betweenness and degree of all nodes, +1 SD refers to values of betweenness and degree above one standard deviations (+1 SD), +2 SD refers to values of betweenness and degree above two standard deviations (+2 SD).

**Table 4:** Topological parameters of CHD CePIN after candidate targets removal.

Removed Node	Category	Hub/Bottleneck	Shortest Paths	Characteristic Path Length	Network Diameter
EDN1	shared	hub-bottleneck	988 (38%)	3.332	7
FGG	unique	bottleneck	1238 (48%)	3.313	7
SLC9A3	unique	bottleneck	1232 (50%)	3.344	7
STAT3	shared	bottleneck	1310 (51%)	3.382	7
KNG1	shared	hub	1828 (71%)	4.658	11
JUN	shared	hub	1934 (75%)	5.411	12
without removing	-	-	2652 (100%)	5.572	13

**Table 5:** Gene Ontology (GO) biological process terms of the CHD CePIN modules.

Module	*p*-Value	Description
1	7.76 × 10^−9^	inflammatory response
2	4.89 × 10^−7^	G-protein-coupled receptor signaling pathway
3	4.91 × 10^−11^	regulation of cell cycle
4	5.23 × 10^−11^	heme catabolic process
5	6.08 × 10^−8^	regulation of I-κB kinase/NF-κB signaling
6	4.89 × 10^−9^	blood coagulation
7	2.12 × 10^−10^	arachidonic acid metabolic process
8	1.42 × 10^−6^	regulation of blood volume by renin-angiotensin
9	1.51 × 10^−7^	G-protein-coupled receptor signaling pathway

Nuclear factor kappa-light-chain-enhancer of activated B cells (NF-κB); Inhibitor of nuclear factor kappa-B kinase (I-κB).

## Supplementary Materials

Supplementary materials can be found at www.mdpi.com/1422-0067/18/6/1298/s1.

## Abbreviations

CHD	coronary heart disease
PPIs	protein–protein interactions
TCM	traditional Chinese medicine
DSS	Danshensu
PIN	protein–protein interaction network
CePIN	coexpression protein interaction network
CePPIs	coexpression protein-protein interactions
GO	Gene Ontology
