# Supplementary material for: Using Coexpression Protein Interaction Network Analysis to Identify Mechanisms of Danshensu Affecting Patients with Coronary Heart Disease
Source: Int J Mol Sci. 2017 Jun 19;18(6):1298. doi: 10.3390/ijms18061298 (PMC5486119; doi:10.3390/ijms18061298)
Supplement: Supplementary file 1 [file ijms-18-01298-s001.pdf]

Supplementary Data for

Using Coexpression Protein Interaction Network Analysis to Identify Mechanisms of Danshensu Affecting Patients with Coronary Heart Disease

Mengqi Huo, Zhixin Wang, Dongxue Wu, Yanling Zhang*, Yanjiang Qiao*

(Key Laboratory of Traditional Chinese Medicine Information Engineer of State Administration of Traditional Chinese Medicine; School of Chinese Material Medica, Beijing University of Chinese Medicine, Beijing 100102, China)

Correspondence should be addressed to Yanling Zhang; zhangyanling@bucm.edu.cn
Correspondence should be addressed to Yanjiang Qiao; yjqiao@bucm.edu.cn

Contents

Figure:

Fig. S1. PIN of DSS.

Fig. S2. Non-CHD CePIN.

Table:

Table S1 Information of the microarray data

Table S2 Information of CHD samples and non-CHD samples

Table S3 Protein information of CHD CePIN and non-CHD CePIN

Table S4 Nodes with degree and betweenness values in CHD CePIN

Table S5 Comparative analysis of the expression level of genes in the two states

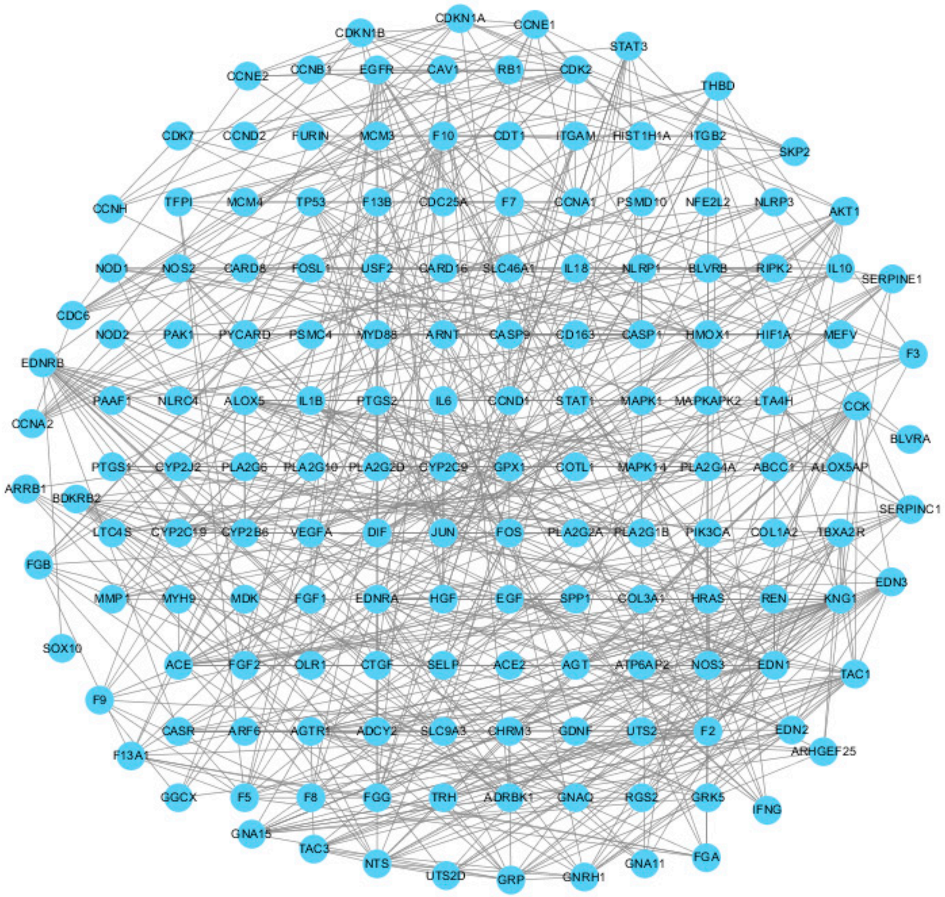

Figure S1. PIN of DSS

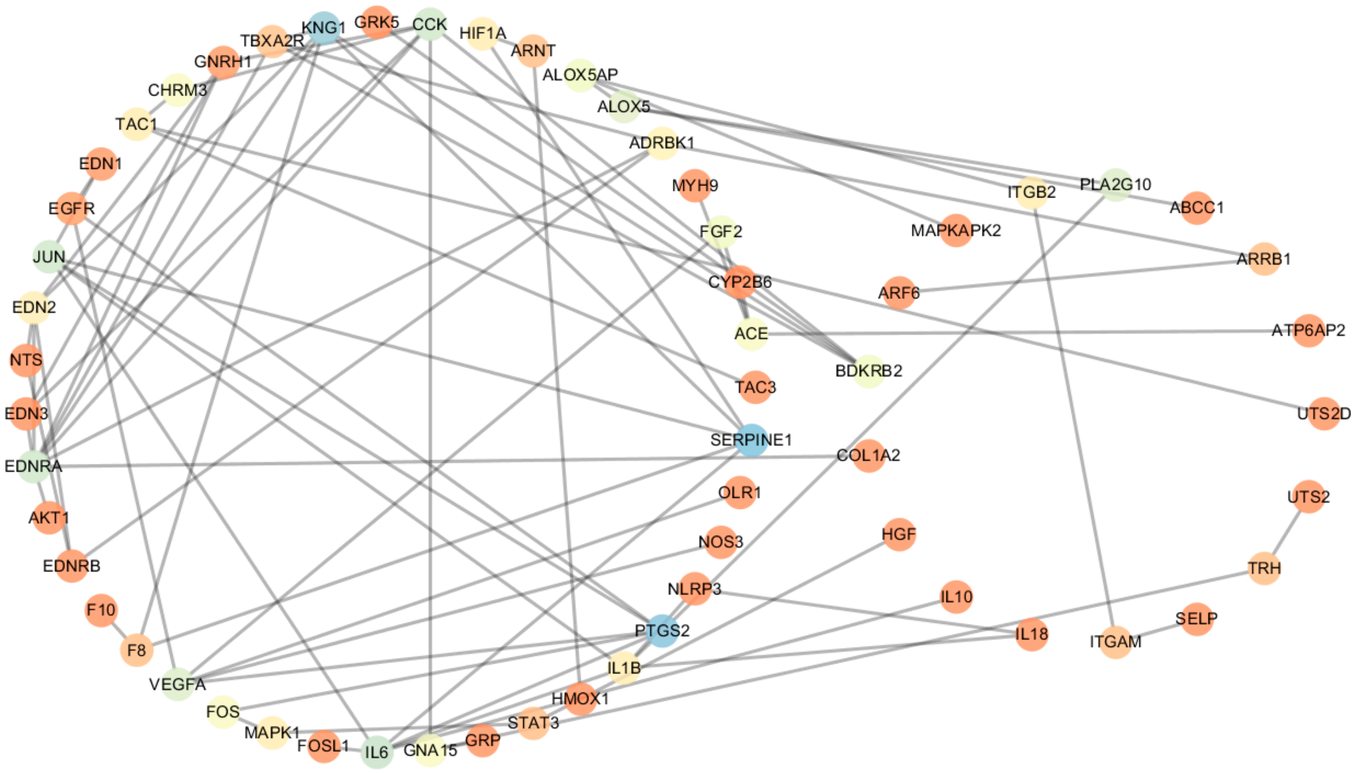

Figure S2. Non-CHD CePIN colored based on the betweenness centrality of each node from least (red) to greatest (blue).

Table S1 Information of the Microarray Data

Item	Description
Series title	Global gene expression profile of coronary artery disease in Asian Indians
Series geo accession	GSE42148
Series status	Public on Nov 09 2012
Series submission date	Nov 08 2012
Series last update date	Jun 23 2016
Series overall design	We selected 13 patients with angiographically confirmed coronary heart disease (CHD) between ages 40–55 years and 11 population-based asymptomatic controls with normal ECG and matched for age, gender and shared risk factors such as diabetes and hypertension to that of the cases. Global gene expression profiling was performed on the Agilent microarray platform.
Series type	Expression profiling by array
Series contributor	Prathima,,Arvind
Series contributor	Jayashree,,Shanker
Series sample id	GSM1033576 GSM1033577 GSM1033578 GSM1033579 GSM1033580 GSM1033581 GSM1033582 GSM1033583 GSM1033584 GSM1033585 GSM1033586 GSM1033587 GSM1033588 GSM1033589 GSM1033590 GSM1033591 GSM1033592 GSM1033593 GSM1033594 GSM1033595 GSM1033596 GSM1033597 GSM1033598 GSM1033599
Series contact name	prathima,,arvind
Series contact email	prathima79@gmail.com
Series contact fax	080-27835302
Series contact department	Functional Genomics
Series contact institute	Thrombosis Research Institute
Series contact address	#258/A, Bommasandra Industrial Area, Anekal Taluk

Series contact city	Bangalore
Series contact zip/postal code	560099
Series contact country	India
Series supplementary file	ftp://ftp.ncbi.nlm.nih.gov/pub/geo/DATA/supplementary/series/GSE42148/GSE42148 RAW.tar
Series platform id	GPL13607
Series platform taxid	9606
Series sample taxid	9606
Series relation	BioProject: https://www.ncbi.nlm.nih.gov/bioproject/PRJNA179208

Table S2 Information of CHD Samples and Non-CHD Samples

Geo accession	Type	Source name	Organism	Tissue	Gender	Age	Characteristics	Sample description
GSM1033576	RNA	Peripheral Blood, control, 14hrs fasting , replicate 1	Homo sapiens	whole blood	male	50	disease state: control	Gene expression in control samples
GSM1033577	RNA	Peripheral Blood, control, 14hrs fasting , replicate 1	Homo sapiens	whole blood	male	51	disease state: control	Gene expression in control samples
GSM1033578	RNA	Peripheral Blood, control, 14hrs fasting , replicate 1	Homo sapiens	whole blood	male	53	disease state: control	Gene expression in control samples
GSM1033579	RNA	Peripheral Blood, control, 14hrs fasting , replicate 1	Homo sapiens	whole blood	male	52	disease state: control	Gene expression in control samples
GSM1033580	RNA	Peripheral Blood, control, 14hrs fasting , replicate 1	Homo sapiens	whole blood	male	48	disease state: control	Gene expression in control samples
GSM1033581	RNA	Peripheral Blood, control, 14hrs fasting , replicate 1	Homo sapiens	whole blood	male	48	disease state: control	Gene expression in control samples
GSM1033582	RNA	Peripheral Blood, control, 14hrs fasting , replicate 1	Homo sapiens	whole blood	male	46	disease state: control	Gene expression in control samples
GSM1033583	RNA	Peripheral Blood, control, 14hrs fasting , replicate 1	Homo sapiens	whole blood	male	53	disease state: control	Gene expression in control samples
GSM1033584	RNA	Peripheral Blood, control, 14hrs fasting , replicate 1	Homo sapiens	whole blood	male	48	disease state: control	Gene expression in control samples
GSM1033585	RNA	Peripheral Blood, control, 14hrs fasting , replicate 1	Homo sapiens	whole blood	male	55	disease state: control	Gene expression in control samples
GSM1033586	RNA	Peripheral Blood, control, 14hrs fasting , replicate 1	Homo sapiens	whole blood	male	53	disease state: control	Gene expression in control samples

GSM1033587	RNA	Peripheral Blood, case, 14hrs fasting , replicate 1	Homo sapiens	whole blood	male	50	disease state: coronary heart disease	Gene expression in case samples
GSM1033588	RNA	Peripheral Blood, case, 14hrs fasting , replicate 1	Homo sapiens	whole blood	male	50	disease state: coronary heart disease	Gene expression in case samples
GSM1033589	RNA	Peripheral Blood, case, 14hrs fasting , replicate 1	Homo sapiens	whole blood	male	46	disease state: coronary heart disease	Gene expression in case samples
GSM1033590	RNA	Peripheral Blood, case, 14hrs fasting , replicate 1	Homo sapiens	whole blood	male	50	disease state: coronary heart disease	Gene expression in case samples
GSM1033591	RNA	Peripheral Blood, case, 14hrs fasting , replicate 1	Homo sapiens	whole blood	male	41	disease state: coronary heart disease	Gene expression in case samples
GSM1033592	RNA	Peripheral Blood, case, 14hrs fasting , replicate 1	Homo sapiens	whole blood	male	57	disease state: coronary heart disease	Gene expression in case samples
GSM1033593	RNA	Peripheral Blood, case, 14hrs fasting , replicate 1	Homo sapiens	whole blood	male	52	disease state: coronary heart disease	Gene expression in case samples
GSM1033594	RNA	Peripheral Blood, case, 14hrs fasting , replicate 1	Homo sapiens	whole blood	male	43	disease state: coronary heart disease	Gene expression in case samples
GSM1033595	RNA	Peripheral Blood, case, 14hrs fasting , replicate 1	Homo sapiens	whole blood	male	45	disease state: coronary heart disease	Gene expression in case samples
GSM1033596	RNA	Peripheral Blood, case, 14hrs fasting , replicate 1	Homo sapiens	whole blood	male	49	disease state: coronary heart disease	Gene expression in case samples
GSM1033597	RNA	Peripheral Blood, case, 14hrs fasting , replicate 1	Homo sapiens	whole blood	male	51	disease state: coronary heart disease	Gene expression in case samples
GSM1033598	RNA	Peripheral Blood, case, 14hrs fasting , replicate 1	Homo sapiens	whole blood	male	48	disease state: coronary heart disease	Gene expression in case samples
GSM1033599	RNA	Peripheral Blood, case, 14hrs fasting , replicate 1	Homo sapiens	whole blood	male	47	disease state: coronary heart disease	Gene expression in case samples

Table S3 Protein Information of CHD CePIN and Non-CHD CePIN

Proteins in non-CHD CePIN	uniprot ID	Proteins in CHD CePIN	uniprot ID
ABCC1	P33527	ACE	P12821
ACE	P12821	ADRBK1	P25098
ADRBK1	P25098	AGT	P01019
AGTR1	P30556	AKT1	P31749
AKT1	P31749	ALOX5	P09917
ALOX5	P09917	ALOX5AP	P20292
ALOX5AP	P20292	ARNT	P27540
ARF6	P62330	BLVRA	P53004
ARNT	P27540	BLVRB	P30043
ARRB1	P49407	CARD8	Q9Y2G2
ATP6AP2	O75787	CASP1	P29466
BDKRB2	P30411	CASP9	P55211
CARD16	Q5EG05	CASR	P41180
CASP1	P29466	CCK	P06307
CASP9	P55211	CCNA1	P78396
CCK	P06307	CCNA2	P20248
CCNA1	P78396	CCND1	P24385
CCNA2	P20248	CCND2	P30279
CCND2	P30279	CCNE1	P24864
CCNE1	P24864	CCNH	P51946
CCNE2	O96020	CD163	Q86VB7
CDC25A	P30304	CDC25A	P30304

CDC6	Q99741	CDK2	P24941
CDKN1A	P38936	CDK7	P50613
CDKN1B	P46527	CDKN1A	P38936
CDT1	Q9H211	CDKN1B	P46527
CHRM3	P20309	CDT1	Q9H211
COL1A2	P08123	CHRM3	P20309
CYP2B6	P20813	CYP2B6	P20813
CYP2C19	P33261	EDN1	P05305
EDN1	P05305	EDN2	P20800
EDN2	P20800	EDN3	P14138
EDN3	P14138	EDNRA	P25101
EDNRA	P25101	EDNRB	P24530
EDNRB	P24530	EGFR	P00533
EGFR	P00533	F10	P00742
F10	P00742	F13A1	P00488
F3	P13726	F13B	P05160
F5	P12259	F2	P00734
F8	P00451	F3	P13726
F9	P00740	F5	P12259
FGF2	P09038	F8	P00451
FOS	P01100	F9	P00740
FOSL1	P15407	FGB	P02675
FURIN	P09958	FGF1	P05230
GNA15	P30679	FGF2	P09038
GNAQ	P50148	FGG	P02679
GNRH1	P01148	FOS	P01100

GRK5	P34947	FOSL1	P15407
GRP	P07492	FURIN	P09958
HGF	P14210	GDNF	P39905
HIF1A	Q16665	GNA11	P29992
HMOX1	P09601	GNA15	P30679
IL10	P22301	GNAQ	P50148
IL18	Q14116	GNRH1	P01148
IL1B	P01584	GRK5	P34947
IL6	P05231	GRP	P07492
ITGAM	P11215	HIF1A	Q16665
ITGB2	P05107	HIST1H1A	Q02539
JUN	P05412	HMOX1	P09601
KNG1	P01042	IL1B	P01584
MAPK1	P28482	IL6	P05231
MAPKAPK2	P49137	JUN	P05412
MYD88	Q99836	KNG1	P01042
MYH9	P35579	LTA4H	P09960
NLRC4	Q9NPP4	MAPK1	P28482
NLRP3	Q96P20	MAPK14	Q16539
NOD1	Q9Y239	MDK	P21741
NOD2	Q9HC29	MEFV	O15553
NOS3	P29474	MYD88	Q99836
NTS	P30990	MYH9	P35579
OLR1	P78380	NLRC4	Q9NPP4
PIK3CA	P42336	NLRP3	Q96P20
PLA2G10	O15496	NOD2	Q9HC29

PLA2G1B	P04054	NOS3	P29474
PTGS2	P35354	NTS	P30990
RGS2	P41220	OLR1	P78380
SELP	P16109	PIK3CA	P42336
SERPINC1	P01008	PLA2G1B	P04054
SERPINE1	P05121	PLA2G2D	Q9UNK4
STAT1	P42224	PLA2G4A	P47712
STAT3	P40763	PLA2G6	O60733
TAC1	P20366	PSMC4	P43686
TAC3	Q9UHF0	PSMD10	O75832
TBXA2R	P21731	PTGS1	P23219
THBD	P07204	PTGS2	P35354
TP53	P04637	RB1	P06400
TRH	P20396	REN	P00797
UTS2	O95399	RGS2	P41220
UTS2D	Q765I0	SERPINC1	P01008
VEGFA	P15692	SERPINE1	P05121
——	——	SLC46A1	Q96NT5
——	——	SLC9A3	P48764
——	——	STAT1	P42224
——	——	STAT3	P40763
——	——	TAC1	P20366
——	——	TAC3	Q9UHF0
——	——	TBXA2R	P21731
——	——	VEGFA	P15692

Table S4 Nodes with Degree and Betweenness Values in CHD CePIN

Name	Category	Hub/bottleneck	Betweenness	Degree
EDN1	shared	hub-bottleneck	0.63673203[a]	7[b]
FGG	unique	bottleneck	0.51450980[a]	4
SLC9A3	unique	bottleneck	0.49411765[a]	2
STAT3	shared	bottleneck	0.48627451[a]	2
F10	shared	——	0.41058824	5
JUN	shared	hub	0.37490196	7[b]
F8	shared	——	0.32156863	4
KNG1	shared	hub	0.28313725	7[b]
CCND1	unique	——	0.21803922	3
TBXA2R	shared	——	0.15137255	5
EGFR	shared	——	0.14287582	5
CDKN1A	shared	——	0.11333333	3
VEGFA	shared	——	0.10000000	6
PTGS2	shared	——	0.08535948	6
F13B	unique	——	0.07686275	3
F9	shared	——	0.04352941	4
HIF1A	shared	——	0.04248366	2
TAC3	shared	——	0.03921569	2
IL1B	shared	——	0.03921569	4
GRP	shared	——	0.03921569	4
CCNH	unique	——	0.03921569	2
CCK	shared	——	0.03921569	4
IL6	shared	——	0.02169935	5

CCND2	shared	——	0.01921569	2
CCNA1	shared	——	0.01921569	2
F3	shared	——	0.00222222	2
CDKN1B	shared	——	0.00039200	2
SERPINE1	shared	——	0	2
SERPINC1	shared	——	0	1
RGS2	shared	——	0	1
OLR1	shared	——	0	2
NLRP3	shared	——	0	1
MAPK14	unique	——	0	2
GRK5	shared	——	0	1
GNRH1	shared	——	0	1
GNAQ	shared	——	0	2
GNA15	shared	——	0	1
FURIN	shared	——	0	2
FOSL1	shared	——	0	2
FOS	shared	——	0	1
FGF2	shared	——	0	1
FGF1	unique	——	0	1
FGB	unique	——	0	2
F5	shared	——	0	2
F13A1	unique	——	0	2
EDNRB	shared	——	0	1
EDNRA	shared	——	0	3
CHRM3	shared	——	0	1
CDK7	unique	——	0	1

CASR	unique	——	0	1
AKT1	shared	——	0	2
ADRBK1	shared	——	0	2
Average			0.091432882	2.7307692
1 SD			0.254025973	4.4810116
2 SD			0.416619064	6.2312540

Note: a and b refers to nodes with values of betweenness and degree above two standard deviations (+2 SD) compared with thresholds, respectively.

Table S5 Comparative Analysis of the Expression Level of Genes in the Two States

gene name	ID_REF	category	logFC	AveExpr	P.Value
MYH9	34157	shared	-0.420	8.810	0.002
GNAQ	7210	shared	-0.429	9.044	0.005
GNAQ	9293	shared	-0.370	9.073	0.010
EDN3	7688	shared	0.494	6.458	0.012
HIF1A	56021	shared	-0.750	7.673	0.016
PTGS2	40701	shared	-1.550	10.595	0.018
OLR1	31425	shared	-1.615	5.009	0.018
PTGS2	26834	shared	-1.555	10.636	0.018
PTGS2	29363	shared	-1.523	10.629	0.019
PTGS2	7785	shared	-1.541	10.666	0.019
PTGS2	38313	shared	-1.528	10.644	0.019
PTGS2	34168	shared	-1.513	10.658	0.021
STAT3	44960	shared	-0.485	8.671	0.022
PTGS2	13418	shared	-1.489	10.567	0.025
PTGS2	47835	shared	-1.463	10.602	0.026
PTGS2	50759	shared	-1.451	10.626	0.026
STAT3	35340	shared	-0.469	8.763	0.027
PTGS2	50025	shared	-1.449	10.626	0.027
CCNA1	42352	shared	-0.819	3.069	0.034
AKT1	8124	shared	-0.265	11.912	0.034
EDN3	8352	shared	0.391	6.439	0.035
STAT3	16168	shared	-0.441	8.743	0.040

STAT3	44202	shared	-0.442	8.742	0.044
STAT3	58201	shared	-0.463	10.427	0.045
STAT3	35925	shared	-0.422	8.647	0.047
HIF1A	11140	shared	-0.423	11.422	0.049
STAT3	32141	shared	-0.481	10.372	0.050
STAT3	1574	shared	-0.429	8.755	0.052
ALOX5AP	41412	shared	-0.358	13.829	0.055
ADRBK1	33069	shared	-0.219	14.460	0.059
FOSL1	14997	shared	-0.681	5.319	0.062
STAT3	51525	shared	-0.394	8.684	0.062
ALOX5	8441	shared	-0.313	15.035	0.065
STAT3	51557	shared	-0.391	8.718	0.067
NTS	52654	shared	0.618	2.240	0.067
HIF1A	19927	shared	-0.379	11.461	0.067
AKT1	19519	shared	-0.257	11.937	0.073
VEGFA	31937	shared	-0.815	5.830	0.073
GRP	61769	shared	-0.281	4.798	0.074
ADRBK1	44736	shared	-0.197	14.432	0.077
STAT3	17981	shared	-0.351	8.730	0.084
ALOX5AP	4609	shared	-0.333	13.758	0.085
CCND2	57804	shared	0.358	11.242	0.085
OLR1	19187	shared	-1.259	5.099	0.085
PLA2G1B	39317	shared	0.266	6.199	0.086
STAT3	52311	shared	-0.366	8.742	0.086

ALOX5	38072	shared	-0.307	14.994	0.089
MYH9	22113	shared	-0.256	14.111	0.092
PIK3CA	9068	shared	-0.233	9.055	0.095
HIF1A	37966	shared	-0.339	11.470	0.096
GNRH1	49670	shared	0.431	6.138	0.098
NOS3	7690	shared	0.240	8.799	0.099
PLA2G1B	33498	shared	0.358	6.210	0.100
FGF2	10668	shared	-0.261	1.974	0.103
TAC3	59778	shared	-0.371	3.606	0.103
MYD88	41595	shared	-0.320	12.917	0.107
FOS	33226	shared	-0.934	12.868	0.110
HIF1A	33770	shared	-0.339	11.473	0.110
ALOX5	24874	shared	-0.274	15.025	0.113
FOS	60096	shared	-0.939	12.893	0.113
ALOX5	55136	shared	-0.278	15.011	0.113
CDKN1B	62789	shared	-0.311	11.802	0.116
PIK3CA	27075	shared	-0.231	9.057	0.118
NLRP3	36170	shared	-0.797	12.791	0.119
RGS2	10299	shared	-0.301	16.993	0.120
GRP	59696	shared	-0.319	4.828	0.124
NLRP3	22438	shared	-0.858	10.661	0.124
SERPINC1	9562	shared	-0.637	3.417	0.125
SERPINE1	55351	shared	-0.580	5.212	0.126
ALOX5	28693	shared	-0.270	15.026	0.128

HIF1A	13714	shared	-0.324	11.430	0.128
NLRC4	11683	shared	-0.652	3.352	0.129
HIF1A	35656	shared	-0.303	11.448	0.131
NLRP3	45014	shared	-0.774	12.747	0.137
HIF1A	33512	shared	-0.307	11.433	0.139
CYP2B6	12149	shared	0.403	7.577	0.139
HIF1A	47991	shared	-0.313	11.416	0.144
ALOX5	22176	shared	-0.263	14.962	0.145
ALOX5	56443	shared	-0.251	14.999	0.147
TAC1	53472	shared	0.566	2.308	0.148
GNRH1	11887	shared	0.318	6.152	0.149
FURIN	49158	shared	-0.331	8.183	0.150
NLRC4	54759	shared	0.427	10.836	0.150
NLRC4	44711	shared	0.402	10.790	0.152
MYD88	2931	shared	-0.323	12.863	0.154
SERPINC1	8700	shared	-0.531	2.944	0.155
NLRC4	26055	shared	0.409	10.817	0.158
RGS2	56907	shared	-0.251	16.917	0.158
EGFR	42170	shared	-0.355	2.652	0.159
NLRC4	32266	shared	0.395	10.834	0.161
NLRC4	50138	shared	0.414	10.807	0.166
NLRC4	52784	shared	0.400	10.829	0.169
MYH9	49568	shared	-0.205	14.141	0.170
HIF1A	12200	shared	-0.307	11.373	0.173

VEGFA	58097	shared	-0.656	5.826	0.173
ALOX5	25550	shared	-0.242	14.979	0.176
CCNA2	47060	shared	-0.400	5.036	0.177
HIF1A	21264	shared	-0.292	11.414	0.180
GRK5	18422	shared	0.231	11.633	0.182
CHRM3	1005	shared	0.402	4.366	0.183
RGS2	23984	shared	-0.250	16.999	0.191
ALOX5	58349	shared	-0.226	15.055	0.192
NLRC4	29448	shared	0.381	10.923	0.194
NOS3	52200	shared	0.166	8.717	0.201
EGFR	28812	shared	0.313	2.247	0.202
CDKN1B	20485	shared	0.193	11.963	0.208
ACE	24532	shared	0.223	8.049	0.210
CYP2B6	47702	shared	0.339	7.560	0.212
FOSL1	26405	shared	-0.427	5.405	0.213
NLRC4	35374	shared	0.352	10.837	0.215
F3	17727	shared	-1.215	3.461	0.230
EGFR	22150	shared	0.338	5.431	0.232
RGS2	60719	shared	-0.229	17.024	0.234
GRP	24191	shared	0.217	4.718	0.236
NLRC4	28923	shared	0.345	10.948	0.237
CDKN1B	28432	shared	0.191	11.989	0.238
NLRC4	39855	shared	0.313	10.835	0.248
CCND2	31861	shared	0.235	11.172	0.248

TBXA2R	46133	shared	0.293	9.671	0.250
F5	50909	shared	-0.295	8.598	0.262
ALOX5	55087	shared	-0.199	15.027	0.264
CDKN1B	61887	shared	0.159	11.962	0.265
SERPINE1	12979	shared	0.635	5.556	0.270
RGS2	16210	shared	-0.212	16.975	0.274
TBXA2R	43300	shared	0.177	9.964	0.277
CCNA1	24007	shared	-0.509	2.730	0.277
RGS2	2036	shared	-0.194	16.951	0.279
FURIN	60036	shared	-0.269	7.984	0.289
CASP9	45621	shared	-0.132	9.859	0.292
SERPINC1	27301	shared	-0.499	3.542	0.292
JUN	34636	shared	-0.615	8.492	0.293
CCNA2	55341	shared	-0.254	5.042	0.294
TBXA2R	32252	shared	0.376	10.278	0.295
EDN2	14983	shared	-0.585	3.597	0.296
CCNA2	40009	shared	-0.254	5.108	0.307
CDKN1A	41195	shared	-0.316	6.511	0.313
CASP1	26598	shared	-0.166	14.020	0.313
CCNA2	17983	shared	-0.251	5.145	0.314
RGS2	53342	shared	-0.197	16.980	0.315
SERPINC1	20178	shared	-0.320	3.273	0.327
CDKN1B	47763	shared	0.147	11.911	0.332
CCNA2	56468	shared	-0.263	5.077	0.333

F5	56037	shared	-0.262	8.599	0.338
CDKN1B	27926	shared	0.138	11.964	0.338
CDKN1B	53165	shared	0.142	11.919	0.347
GNA15	5492	shared	-0.225	8.456	0.348
EGFR	31340	shared	-0.234	2.285	0.349
RGS2	57466	shared	-0.174	16.995	0.349
SERPINE1	39108	shared	0.293	4.956	0.352
FGF2	45242	shared	-0.251	2.658	0.357
F3	7738	shared	-1.036	3.749	0.361
CDKN1B	20903	shared	0.138	12.007	0.369
TBXA2R	42546	shared	0.231	9.679	0.371
GNA15	17140	shared	-0.211	8.446	0.372
CASP9	49226	shared	-0.108	9.863	0.372
NTS	25565	shared	0.235	2.219	0.373
CASP1	35013	shared	-0.138	14.077	0.373
JUN	16213	shared	-0.513	8.531	0.375
CDC25A	53584	shared	0.342	5.453	0.375
MAPK1	34283	shared	-0.137	11.968	0.378
F10	45032	shared	0.369	4.696	0.397
STAT1	9864	shared	-0.200	11.616	0.399
TAC1	46240	shared	0.156	2.049	0.401
F10	45029	shared	0.314	4.618	0.408
GRP	3468	shared	0.178	5.203	0.420
SERPINC1	26344	shared	-0.264	3.479	0.422

GRP	58602	shared	-0.185	4.988	0.426
EGFR	35341	shared	0.271	2.449	0.431
EGFR	48315	shared	-0.445	2.885	0.434
CCNA2	16593	shared	-0.213	5.001	0.436
FGF2	48599	shared	0.220	2.359	0.439
CHRM3	43688	shared	-0.164	4.076	0.439
MAPK1	56916	shared	-0.116	11.984	0.441
CCNA2	29627	shared	-0.180	5.106	0.456
GRP	51738	shared	-0.125	4.991	0.460
GRP	43925	shared	-0.131	5.149	0.461
STAT1	43328	shared	-0.177	11.562	0.466
STAT1	39506	shared	-0.167	11.607	0.495
GRK5	29310	shared	0.122	11.683	0.495
EDNRB	35764	shared	-0.292	3.238	0.498
NOD2	45083	shared	0.195	11.723	0.503
EDN1	38025	shared	-0.169	5.671	0.510
SERPINC1	25741	shared	-0.237	3.337	0.515
CCNA2	60197	shared	-0.180	5.143	0.526
STAT1	5417	shared	-0.160	11.586	0.527
EDN1	41571	shared	-0.276	6.903	0.535
EDN2	31812	shared	-0.206	3.575	0.541
STAT1	18450	shared	-0.144	11.671	0.546
EDN1	50788	shared	-0.262	6.832	0.550
STAT1	24223	shared	-0.149	11.575	0.552

IL6	60427	shared	-0.457	6.387	0.554
EGFR	59735	shared	-0.152	2.464	0.555
SERPINC1	31399	shared	0.164	3.836	0.557
MAPK1	35756	shared	-0.093	11.958	0.573
CDT1	46280	shared	-0.145	7.432	0.575
RGS2	6559	shared	-0.106	16.888	0.578
EDN1	44328	shared	-0.243	6.842	0.579
NOD2	29150	shared	0.160	11.699	0.582
STAT1	28078	shared	-0.134	11.550	0.582
EDN1	6709	shared	-0.233	6.878	0.592
EGFR	40016	shared	-0.130	5.466	0.592
EDN1	18897	shared	-0.232	6.866	0.597
EDN1	23339	shared	-0.228	6.843	0.600
GRP	27262	shared	0.114	4.948	0.602
TBXA2R	27843	shared	0.142	9.807	0.603
CCK	18982	shared	0.197	6.870	0.612
IL6	59553	shared	-0.378	6.477	0.620
GRP	41155	shared	0.082	5.275	0.622
VEGFA	34225	shared	-0.184	6.312	0.625
CDKN1B	42185	shared	0.067	11.884	0.640
STAT1	31396	shared	-0.108	11.667	0.640
ARNT	53869	shared	-0.080	8.083	0.641
IL6	59476	shared	-0.358	6.401	0.643
EGFR	1625	shared	0.120	2.861	0.651

STAT1	52577	shared	-0.109	11.589	0.653
MAPK1	51084	shared	-0.067	11.989	0.665
CDKN1A	20633	shared	-0.152	6.354	0.673
EDNRA	54655	shared	-0.108	5.908	0.673
HMOX1	10436	shared	-0.099	14.052	0.677
EDN3	55357	shared	0.190	2.900	0.682
CDC25A	32173	shared	0.064	5.766	0.684
CCNA2	198	shared	0.149	4.956	0.685
SERPINE1	15225	shared	0.101	5.800	0.686
EDNRB	58740	shared	0.163	3.167	0.692
HMOX1	6137	shared	-0.093	14.106	0.695
EDN1	52369	shared	-0.171	6.856	0.696
ARNT	56709	shared	-0.065	8.091	0.700
EDN1	47998	shared	-0.168	6.849	0.704
SERPINE1	27305	shared	0.149	4.986	0.709
CDT1	24142	shared	-0.093	7.467	0.716
IL6	35727	shared	-0.274	6.542	0.717
SERPINE1	11725	shared	-0.127	5.340	0.725
STAT1	50347	shared	-0.084	11.574	0.731
MAPK1	42757	shared	-0.054	11.982	0.734
F8	13808	shared	0.149	7.250	0.736
SERPINE1	14259	shared	-0.084	5.780	0.737
MAPK1	1604	shared	0.053	11.905	0.741
HMOX1	7321	shared	-0.073	14.069	0.748

KNG1	27398	shared	-0.056	5.230	0.754
HMOX1	38058	shared	-0.074	14.107	0.758
EDNRA	48767	shared	-0.063	5.743	0.761
MAPK1	60456	shared	-0.047	11.975	0.763
IL6	58400	shared	-0.228	6.553	0.764
IL6	51118	shared	-0.225	6.469	0.769
SERPINC1	2191	shared	-0.132	3.292	0.774
IL6	31128	shared	-0.209	6.562	0.782
IL1B	27208	shared	0.168	13.740	0.790
SERPINE1	43188	shared	-0.114	5.605	0.793
SERPINE1	42150	shared	-0.147	5.551	0.797
MAPK1	17705	shared	-0.041	11.945	0.799
F9	7658	shared	-0.037	1.845	0.801
HMOX1	8174	shared	-0.059	14.092	0.803
RGS2	61456	shared	-0.045	16.936	0.811
IL1B	23193	shared	0.145	13.831	0.816
MAPK1	19426	shared	-0.036	12.051	0.822
EDN1	10255	shared	-0.060	5.443	0.825
CCNA2	22671	shared	-0.057	5.047	0.831
F9	10378	shared	0.087	2.076	0.837
EGFR	50787	shared	0.065	2.491	0.838
EGFR	51758	shared	-0.086	2.476	0.840
GRP	56769	shared	-0.035	5.212	0.844
EGFR	8281	shared	0.036	5.410	0.848

CDKN1B	10391	shared	-0.084	9.372	0.856
MAPK1	27536	shared	-0.028	11.967	0.860
F8	12829	shared	0.079	7.193	0.860
IL1B	12669	shared	0.104	13.734	0.867
IL1B	57253	shared	0.098	13.774	0.873
IL6	12811	shared	-0.119	6.534	0.876
CYP2B6	57579	shared	-0.038	6.824	0.890
SERPINE1	29069	shared	-0.069	5.489	0.892
IL6	57897	shared	-0.104	6.531	0.893
SERPINC1	53933	shared	-0.048	3.425	0.897
IL1B	30444	shared	0.069	13.783	0.912
EDN1	358	shared	0.049	6.828	0.918
IL1B	28590	shared	0.061	13.831	0.921
CCNE1	45950	shared	-0.012	9.221	0.923
ACE	59847	shared	-0.014	8.065	0.923
CCK	61613	shared	-0.042	6.562	0.923
IL1B	38900	shared	0.055	13.723	0.931
IL6	4258	shared	-0.066	6.542	0.931
IL1B	40766	shared	0.051	13.788	0.935
EDNRA	44298	shared	-0.021	6.144	0.935
KNG1	46474	shared	0.008	4.995	0.947
EGFR	22382	shared	-0.023	2.148	0.949
IL1B	6418	shared	0.034	13.763	0.956
TAC3	37970	shared	-0.014	3.501	0.964

HMOX1	47490	shared	-0.010	14.075	0.965
HMOX1	23453	shared	-0.010	14.097	0.965
HMOX1	47891	shared	0.009	14.087	0.968
SERPINC1	40665	shared	0.011	3.591	0.974
HMOX1	28067	shared	-0.007	14.095	0.976
EGFR	49026	shared	-0.004	5.747	0.980
CCNE1	10368	shared	-0.003	9.211	0.980
IL1B	39525	shared	0.013	13.666	0.984
CDKN1B	46424	shared	-0.002	11.842	0.989
HMOX1	18651	shared	-0.003	14.027	0.991
EDN1	12436	shared	-0.002	6.926	0.996
